# Potential field reconstruction-based path planning system for autonomous vehicle with enhancing stability

**DOI:** 10.1371/journal.pone.0333457

**Published:** 2025-09-30

**Authors:** Zihao Fu, Zhixian Liu, Weihua Tan, Kuineng Chen

**Affiliations:** 1 Huaihua University, Huaihua, China; 2 School of Information and Electrical Engineering, Hunan University of Science and Technology, Xiangtan, China; 3 Sanya Institute of Hunan University of Science and Technology, Sanya, China; 4 Hunan Vocational Institute of Technology, Xiangtan, China; National University of Singapore, SINGAPORE

## Abstract

In recent years, the critical role of path planning and path tracking in autonomous vehicles (AV) has led to a growing focus on these areas. Generally speaking, the path planning layer plans the collision-free path, and then the path tracking layer ensures the stability of AV. However, in emergency scenario, the path planned by conventional method is difficult to guarantee the stability of AV. In this study, a potential field reconstruction-based path planning system (PFR-BPPS) is proposed to address the aforementioned issue. The PFR-BPPS consists of two main components: a potential field reconstruction module and an adaptive fusion module. To ensure simultaneous obstacle avoidance and stability for AV, the potential field reconstruction module reconstructs both the potential velocity field and the potential stability field. For adaptive integration of these two fields, the adaptive fusion module employs fuzzy inference rules for effective fusion. The simulation is carried out on the Matlab-Carsim co-simulation platform. The results demonstrate that the path planned by PFR-BPPS exhibits outstanding performance in improving velocity adaptability and maintaining path stability in emergency situations.

## 1 Introduction

With the development of technology, AV has received more and more attention [1,2]. Among the various functions of autonomous vehicles, the obstacle avoidance and stability are both core techniques. In general, the stability of vehicle is discussed in path tracking or motion control. A polytopic model-based robust predictive control scheme is proposed for ensuring path-tracking accuracy and the stability of vehicle operation [3]. A multi-agent system based distributed control architecture together with a hierarchical controller is proposed for the connected and automated vehicles (CAVs) cooperation control system. In this method, artificial potential field (APF) and model predictive control (MPC) are combined to achieve the longitudinal, lateral and yaw motion control of CAVs [4]. The influence of the planned path itself on stability has not been discussed in depth [5]. If the planned path does not accommodate the vehicle stability, especially in emergency scenario, it is difficult to ensure the vehicle stability during the path tracking.

In terms of path planning, there are four main types of methods, grid-based method, sampling-based method, APF method, and velocity obstacle (VO) method. The early works mainly have used graph search based planner to generate shortest path on the graph constructed by the discretization of the environment. The most typical method applied to AV is grid-based method including the state lattice algorithm [6], A* algorithm [7] and Dijkstra algorithm [8]. The largest issue of above methods is that the planned path is discontinuous and the constraint of vehicle dynamic can not be easily considered in the planning, which is obviously not conducive to ensuring the vehicle stability. Sampling-based methods have gained much interest in recent years [9], e.g. probabilistic road maps and rapidly-exploring random trees. They provide the benefit of generating feasible path for both holonomic and non-holonomic systems [10]. However, the resulting path is also discontinuous and therefore jerky. Even with the interpolating curve planners for path smoothing [11–16], the influence of planned path on stability is difficult to determine. APF method is a popular ad hoc approach for solving path planning problem, because of it’s simplicity, fast execution time, and applicability to several robotic problems [17–25]. However, it is also difficult to consider the constraints of vehicle dynamics in traditional APF method, and the effect of velocity which may be decisive in emergency scenario is usually not taken into account. The method of VO [26] is widely adopted for path planning with moving obstacles, and it is suitable for fast online planning in environment with multiple moving obstacles [27,28]. However, the method of VO usually treats moving object as particle, and therefore, ignores the stability of object.

To sum up, the influence of path on vehicle stability has not been considered in the researches mentioned above, which may cause the instability of AV during path tracking, especially in emergency scenario. In order to solve this problem, both obstacle avoidance and stability should be considered at the same time in the path planning for AV.

The key contributions of this research are outlined as follows:

- The path planned by PFR-BPPS can avoid obstacle and enhance the stability for path tracking at the same time, especially in emergency scenario.
- To adapt to time-varying velocity in emergency scenario, the potential velocity field is designed with the idea of VO and APF.
- To enhance the stability of path, the potential stability field is reconstruct in the framework of stability envelope and APF.
- The adaptive fusion module is developed to dynamically adjust the two potential fields based on the level of urgency.

The remainder of this paper is organized as follows. In Sect 2, the problem statement is described. Sect 3 introduces the design of PFR-BPPS. The simulation is presented in Sect 4. Finally, Sect 5 concludes the paper.

## 2 Problem statement

Owing to the disregard for stability, the planned path might become unstable for path tracking, particularly in emergency situations [29]. Therefore, two problems are considered in this paper: (1) the influence of planned path on stability; (2) the influence of velocity on stability.

### The influence of planned path on stability for AV

In general, the stability of AV is discussed in path tracking. However, if the planned path does not accommodate the vehicle stability, it is difficult to ensure the vehicle stability even it is taken into account in the path tracking. On the other hand, in the existing path planning methods, such as A* [7], APF [25] and MPC [30], vehicle stability has not received much attention. And the MPC has its inherent advantage of multiple-constraint processing, so it is relatively suitable for considering stability. The generic MPC problems are described as follows:

$$\min\sum_{k=0}^{n-1}\left(x^{k^{T}}Q^{k}x^{k}+u^{k^{T}}R^{k}u^{k}\right)+x^{n^{T}}Q^{n}x^{n}$$ (1)

subject to

$$x^{k+1}=A^{k}x^{k}+B^{k}u^{k}+C^{k}\;k=0...n-1$$ (2)

$$W^{k}u^{k}\leq Z^{k}\;k=0...n-1$$ (3)

$$H^{k}u^{k}\leq G^{k}\;k=1...n-1$$ (4)

where vehicle input *u* and states *x* are defined at each discrete time step *k* in the prediction horizon. However, the stability of path has been not considered when MPC is used for path planning.

### The influence of velocity in emergency scenario

In emergency scenario, the velocity is a key factor for the safety of path planning. Therefore, the velocity of obstacle and AV should be fully considered for improving the safety of path planning. However, the impact of velocity is seldom considered in traditional approaches [6–8]. For instance, the potential function of the repulsive field in the conventional APF approach is typically expressed as [29]:

$$U_{rep}=\;\left\{ \begin{array}{cc} 0.5\cdot \eta_{u}{\left(\frac{1}{\rho \left(q,q_{obs}\right)}-\frac{1}{\rho_{0}}\right)}^{2} & \rho \left(q,q_{obs}\right)\leq \rho_{0}\\ 0 & \rho \left(q,q_{obs}\right)>\rho_{0} \end{array}\right.$$ (5)

where $\eta_{u}$ represents the strength coefficient. $\rho \left(q,q_{obs}\right)$ represents the distance between obstacle and AV. $\rho_{0}$ denotes the influence radius of obstacle.

As demonstrated in the equation above, the conventional APF method generally establishes the potential field by considering the position of obstacles while neglecting the influence of velocity. This oversight could potentially lead to unsuccessful obstacle avoidance.

## 3 The design of PFR-BPPS

As described earlier, the present methods of path planning do not consider the influence of path on vehicle stability. So the planned path may not accommodate the vehicle stability, and it is difficult to ensure the vehicle stability during the path tracking, especially in emergency scenario.

**Fig 1:**
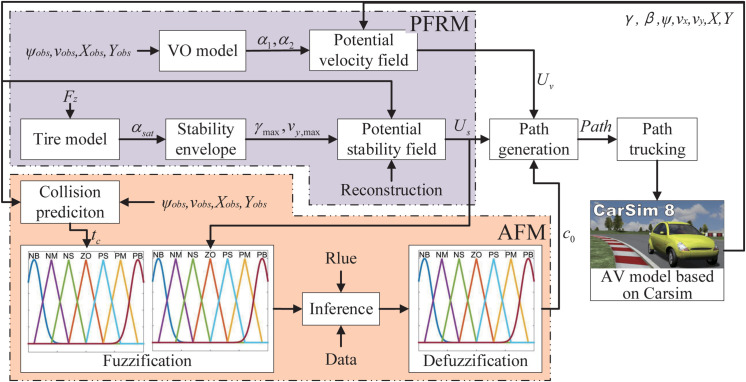
The structural schematic of the PFR-BPPS.

To tackle this challenge, this study introduces a potential field reconstruction-based path planning system (PFR-BPPS). The structural diagram of the PFR-BPPS is illustrated in Fig 1. The PFR-BPPS is composed of two key components: the potential field reconstruction module (PFRM) and the adaptive fusion module (AFM). In order to guarantee the obstacle avoidance and enhance stability for path tracking at the same time, the potential field reconstruction module is designed through reconstructing the potential velocity field and potential stability field. An adaptive fusion module is designed through fuzzy inference rules to realize the adaptive fusion of the two potential fields. By leveraging the fused potential field, an MPC can be employed to generate a path that ensures effective obstacle avoidance while enhancing the stability of path tracking. The definitions of the parameters in Fig 1 are explained in Table 1. This paper places emphasis on path planning, while the path tracking is accomplished via a model predictive controller as detailed in [31].

**Table 1:** The definition of parameter.

Parameter	Definition
*X*, *Y*	The longitudinal position and lateral position of AV
$v_{x}$, $v_{y}$	The longitudinal velocity and lateral velocity of AV
*γ*	The yaw rate of AV
*β*	The side slip angle of AV
*ψ*	The yaw angle of AV
*X*_*obs*_, *Y*_*obs*_	The longitudinal position and lateral position of obstacle
$\psi_{obs}$	The yaw angle of obstacle
$v_{obs}$	The velocity of obstacle
$\alpha_{1}$, $\alpha_{2}$	The shape parameters of the VO model
$U_{v}$, *U*_*s*_	The potential velocity field and potential stability field
*F*_*z*_	The vertical load on the tire
$\gamma_{max}$	The maximum yaw rate obtained by stability envelope
$v_{y,max}$	The maximum lateral velocity obtained by stability envelope
*c*_0_	The adaptive parameter for adjusting two potential fields
$\alpha_{sat}$	The saturating tire slip angle
*t*_*c*_	The predicted collision time

### The design of PFRM

The PFRM is designed to guarantee the obstacle avoidance and enhance stability for path tracking at the same time. To achieve obstacle avoidance in emergency scenario, the ideal of APF and VO are combined to design a new potential velocity field which is constructed by velocity information, and the potential velocity field can adapt to the velocity better than the traditional APF method.

**Fig 2:**
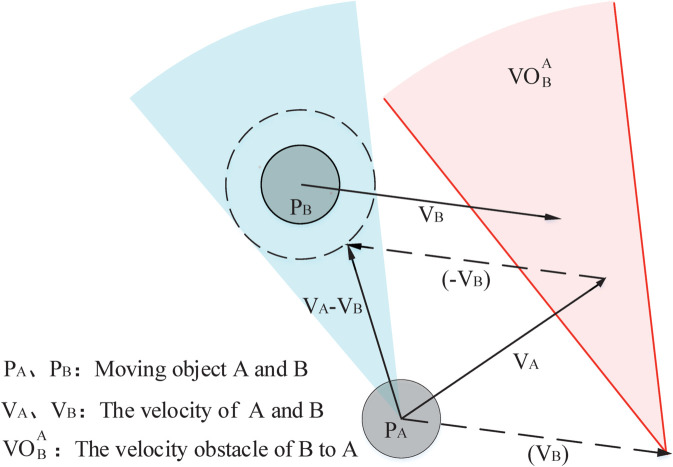
The velocity obstacle.

The method of VO is widely adopted for path planning in a dynamic environment and it’s principle [32] which is illustrated in Fig 2 describes the relativity between collision and velocity. In Fig 2A and 2B are two moveable objects. *P*_*A*_ and *P*_*B*_ denote the position of them, respectively. $V_{A}$ and $V_{B}$ represent the velocities of them. The safe area without collision is represented by the dotted circle. The green region is obtained from the dotted circle and the position of A. $VO_{B}^{A}$ is gained through translating the green region along $V_{B}$. It can be seen that A will collide with B at some time if $V_{A}$ is included in $VO_{B}^{A}$ and A will never collide with B if $V_{A}$ is not included in $VO_{B}^{A}$. Therefore, $VO_{B}^{A}$ is defined as the VO of A to B.

**Fig 3:**
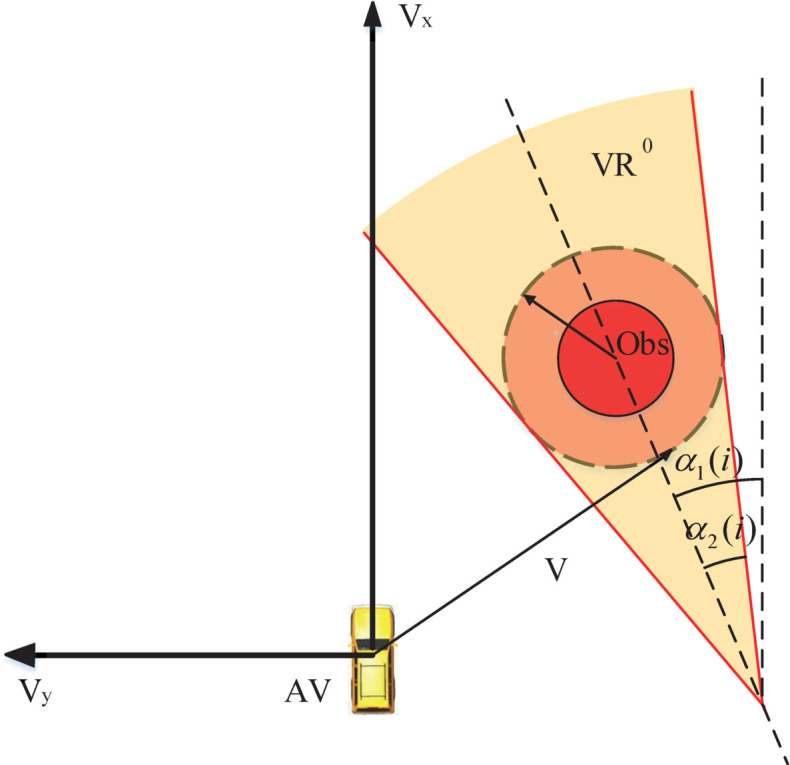
The model of velocity region.

On the basis of VO, the velocity region model can be built as shown in Fig 3. In this model, $VO_{B}^{A}$ is seen as *VR*^0^. $\alpha_{1}$ represents the azimuth angle of obstacle. $\alpha_{2}$ denotes the half-angle of *VR*^0^. $\alpha_{1}$ and $\alpha_{2}$ are defined as follows.

$$\alpha_{1}\left(i\right)=\arctan\frac{Y_{obs}\left(i\right)-Y_{v}}{X_{obs}\left(i\right)-X_{v}}$$ (6)

$$\alpha_{2}\left(i\right)=\arcsin\frac{R\left(i\right)}{\sqrt{{\left(X_{obs}\left(i\right)-X_{v}\right)}^{2}+{\left(Y_{obs}\left(i\right)-Y_{v}\right)}^{2}}}$$ (7)

where the subscript *i* denotes the *i*th obstacle. The safe distance between the obstacle and AV is *R*. $Y_{v}$ and $X_{v}$ are the lateral and longitudinal coordinates of AV, respectively. *Y*_*obs*_ and *X*_*obs*_ represent the lateral and longitudinal coordinates of obstacle, respectively. When the relative velocity between the AV and the obstacle lies outside *VR*^0^, a collision will not occur. By leveraging the velocity region model, one can establish a potential velocity field, and the center line of *VR*^0^ can be formulated as:

$$V_{x}\left(\cdot \right)-V_{obsx}+V_{y}\left(\cdot \right)/\tan\left(\alpha_{1}\left(i\right)\right)-V_{obsy}/\tan\left(\alpha_{1}\left(i\right)\right)=0$$ (8)

where $V_{obsy}$ and $V_{obsx}$ represent the lateral and longitudinal velocity of obstacle, respectively. $V_{y}\left(\cdot \right)$ and $V_{x}\left(\cdot \right)$ denote the coordinates in coordinate system. The distance between the center line and the point in the coordinate system is expressed as:

$$d_{v}=\frac{|V_{x}\left(\cdot \right)-V_{obsx}+\left(V_{y}\cdot -V_{obsy}\right)/\tan\left(\alpha_{1}\left(i\right)\right)|}{\sqrt{\cot^{2}\left(\alpha_{1}\left(i\right)\right)+1}}$$ (9)

**Fig 4:**
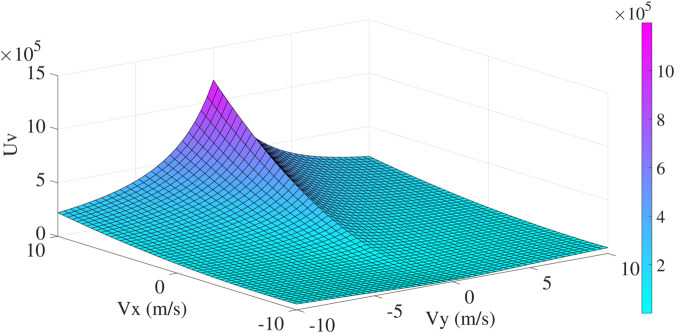
The potential velocity field.

In order to keep the vehicle away from the velocity region, the potential velocity field is constructed as:

$$U_{v}=\left\{ \begin{array}{cc} c_{2}\left({\left(\frac{D_{v}+c_{1}}{d_{v}+c_{1}}\right)}^{2}-1\right) & d_{v}\leq D_{v}\\ \;0 & d_{v}>D_{v} \end{array}\right.$$ (10)

where $D_{v}$ is the width of velocity region at the point ($V_{y}\left(\cdot \right)$,$V_{x}\left(\cdot \right)$). *c*_1_ is a small positive value for avoiding a zero in the denominator. *c*_2_ denotes the intensity coefficient. The potential velocity field can be observed in Fig 4.

To enhance the stability of path, the potential stability field is reconstruct in the framework of stability envelope and APF.

For tire, the saturating tire slip angle appears when no additional force can be generated, and it is defined as [29]:

$$\alpha_{sat}=\arctan\left(\frac{3\eta \mu F_{z}}{C_{a}}\right)$$ (11)

where *μ* and *C*_*a*_ represent the friction coefficients and tire cornering stiffness determined by experiment. *η* is the derating factor. *Fz* denotes the vertical load on the tire.

The stability envelope, which limits the vehicle’s velocity states $v_{y}$ and *γ* based on the maximum steady-state force, is described in [33]. The limit of lateral velocity is defined by rear tire saturation. Saturation appears at the rear tire saturation slip angle, and it can be converted into a bound of lateral velocity.

$$v_{y,max}=v_{x}\alpha_{sat}+b\gamma$$ (12)

where *b* is the distance between rear axle and center of gravity.

An extra constraint on the yaw rate results in a closed set for the vehicle velocity state, which is determined based on the maximum steady-state condition [29]. So the maximum sustained yaw rate is:

$$\gamma_{max}=min\left(\frac{F_{yf,max}\left(a/b+1\right)}{mv_{x}},\frac{F_{yr,max}\left(b/a+1\right)}{mv_{x}}\right)$$ (13)

where *a* represents the distance from the front axle of AV to the center of gravity of AV. *F*_*yf*,*max*_ and *F*_*yr*,*max*_ denote the maximum lateral force of front and rear axle, respectively.

**Fig 5:**
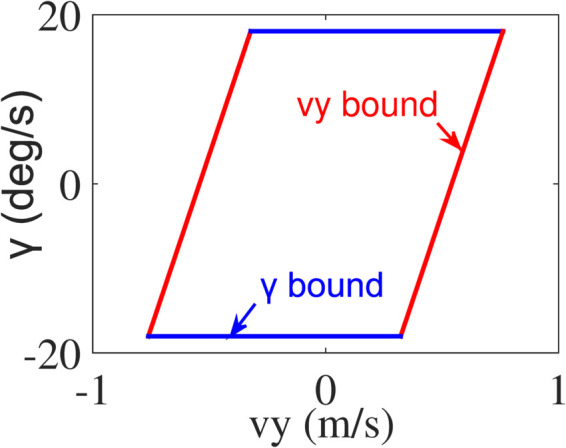
The stability envelope.

The bounds (12) and (13) define an invariant set for vehicle velocity states $v_{y}$ and *γ*, shown in Fig 5. The stability of AV is ensured for all states within the envelope. Exceeding these limits does not automatically lead to instability; however, there is no assurance that a control input can be applied to bring the system closer to the boundary in the next time step when states are outside the envelope [29].

**Fig 6:**
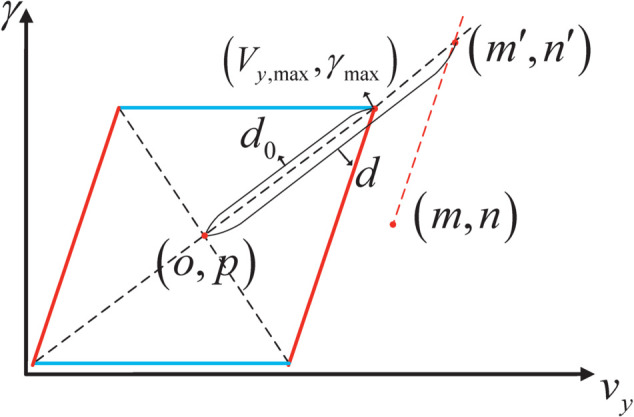
The potential stability model.

To quantify the potential stability field, a novel potential stability model, described in Fig 6, is established. As shown in Fig 6, the center of stability envelope is marked as (o,p). And the red dotted line is parallel to the $v_{y}$ bound. $\left(m^{\prime },n^{\prime }\right)$ is the intersection of red dotted line and the diagonal of stability envelope. $\left(V_{y,max},\gamma_{max}\right)$ is a vertex of the stability envelope. *d* and *d*_0_ correspond to the distance between the two points and (o,p), respectively.

$$d=\sqrt{{\left(m^{\prime }-o\right)}^{2}+{\left(n^{\prime }-p\right)}^{2}}$$ (14)

$$d_{0}=\sqrt{{\left(V_{y,max}-o\right)}^{2}+{\left(\gamma_{max}^{\prime }-p\right)}^{2}}$$ (15)

**Fig 7:**
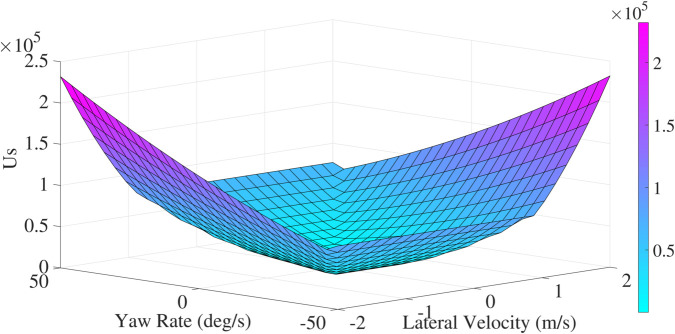
The potential stability field.

The potential stability field of coordinate point (m,n) is denoted as:

$$U_{s}=c_{3}{\left(\frac{d}{d_{0}}\right)}^{2}$$ (16)

where *c*_3_ denotes the margin and intensity coefficient, respectively. And the potential stability field is illustrated in Fig 7.

### The design of AFM

In the AFM, the fuzzy inference is designed to obtain *c*_0_ adaptively for adjusting the size of the two potential fields according to the degree of urgency. The fuzzy inference system mainly includes three steps: fuzzification, inference, defuzzification. And the input variables are *t*_*c*_ and *U*_*s*_, respectively. The output variable is *c*_0_. Initially, the inputs are adjusted to an appropriate scale, and in this study, the scale is defined as [–4, 4].

$$\hat{t_{c}}=-4+8\cdot \frac{t_{c}-Min\left(t_{c}\right)}{Max\left(t_{c}\right)-Min\left(t_{c}\right)}$$ (17)

$$\hat{U_{s}}=-4+8\cdot \frac{U_{s}-Min\left(U_{s}\right)}{Max\left(U_{s}\right)-Min\left(U_{s}\right)}$$ (18)

where the Max(⋅) and Min(⋅) denotes the maximum and minimum, respectively.

**Fig 8:**
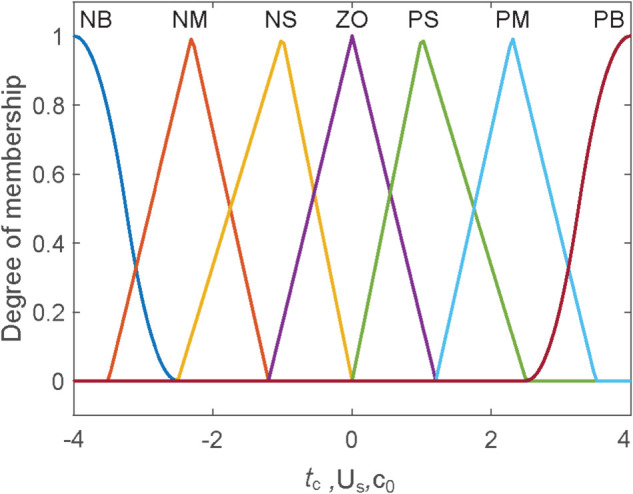
Setting of membership function.

The membership functions are utilized to convert the inputs into fuzzy variables [34]. Subsequently, during the defuzzification process, these fuzzy variables must be transformed back into standard variables. So, some relative relationship functions are adopted for the output. The setting of these membership functions is specifically depicted in Fig 8.

The inference process serves as a crucial step in the fuzzy inference system, where the inference rule must be carefully designed. Based on this requirement, the following principles are employed for constructing the inference rule:

a. When *t*_*c*_ is small, *c*_0_ should take a larger value, whereas when *t*_*c*_ is large, *c*_0_ should take a smaller value;

b. A smaller value of *U*_*s*_ corresponds to a smaller *c*_0_, while a larger *U*_*s*_ results in a larger *c*_0_.

… Specifically, all the rules are presented in Table 2. By means of the fuzzy inference process, the inference result is obtained.

**Table 2:** The inference rules for $c_{0}$.

*c*_0_		*t*_*c*_						
		NB	NM	NS	ZO	PS	PM	PB
*U*_*s*_	NB	PB	PB	PM	PM	PM	PS	ZO
	NM	PB	PM	PM	PM	PS	ZO	NS
	NS	PM	PM	PM	PS	ZO	NS	NM
	ZO	PM	PM	PS	ZO	NS	NM	NM
	PS	PM	PS	ZO	NS	NM	NM	NM
	PM	PS	ZO	NS	NM	NM	NM	NB
	PB	ZO	NS	NM	NM	NM	NB	NB

Finally, the output will be scaled to an appropriate value within the range [0, 1] using the following equation:

$$\hat{c_{0}}=\frac{c_{0}-Min\left(c_{0}\right)}{Max\left(c_{0}\right)-Min\left(c_{0}\right)}$$ (19)

The fused potential field is represented as:

$$U=c_{0}\cdot U_{v}+\left(1-c_{0}\right)\cdot U_{s}$$ (20)

### The path generation

In this work, a MPC is used to generate a path which can guarantee security of the obstacle avoidance and enhance the stability for path tracking.

In the discrete state space model of AV, *x* is considered as the state vector, *u* is used as the control vector and the *y* is chosen as the output vector.

In the discrete state space model of AV, *u* serves as the control vector. *x* represents the state vector. *y* is defined as the output vector.

$$x=[\begin{array}{c} v_{x}\\ v_{y}\\ \psi \\ X\\ Y \end{array}],u=[\begin{array}{c} a_{x}\\ a_{y} \end{array}],y=[\begin{array}{c} \psi_{ref,local}\\ X_{ref,local}\\ Y_{ref,local} \end{array}]$$ (21)

where *Y* and *X* denote the global y-coordinate and x-coordinate of AV, respectively. $v_{y}$ and $v_{x}$ represent the velocity in global y-coordinate and x-coordinate, respectively. *a*_*y*_ and *a*_*x*_ are the acceleration in global y-coordinate and x-coordinate, respectively. *ψ* represents the course angle of AV. Define *k* as the sample time, the discrete state space model is expressed as follows:

where *ψ* denotes the course angle of the AV. *a*_*y*_ and *a*_*x*_ correspond to the acceleration in the global y-coordinate and x-coordinate, respectively. $v_{y}$ and $v_{x}$ indicate the velocity in the global y-coordinate and x-coordinate, respectively. *Y* and *X* represent the global y-coordinate and x-coordinate of the AV, respectively. Let *k* be the sample time; then, the discrete state space model can be formulated as follows:

$$v_{x}\left(k+1\right)=a_{x}\cdot T_{s}+v_{x}\left(k\right)$$ (22)

$$v_{y}\left(k+1\right)=a_{y}\cdot T_{s}+v_{y}\left(k\right)$$ (23)

$$\psi \left(k+1\right)=\omega \cdot T_{s}+\psi \left(k\right)$$ (24)

$$X\left(k+1\right)=\frac{1}{2}\cdot a_{x}\left(k\right)\cdot T_{s}^{2}+v_{x}\left(k\right)\cdot T_{s}+X\left(k\right)$$ (25)

$$Y\left(k+1\right)=\frac{1}{2}\cdot a_{y}\left(k\right)\cdot T_{s}^{2}+v_{y}\left(k\right)\cdot T_{s}+Y\left(k\right)$$ (26)

The predicted state of the AV can be expressed as follows:

$$x\left(k+n|k\right)=f^{k}\left(x\left(k+n-1|k\right),u\left(k+n-1|k\right)\right)\cdot T_{s}\;+x\left(k+n-1|k\right),1\leq n\leq p$$ (27)

where *p* is defined as predictive horizon.

In addition to AV, the state of obstacle should also be considered. *x*_*obs*_ is considered as the state vector of obstacle.

$$x_{obs}={\left[a_{xobs},a_{yobs},v_{xobs},v_{yobs},\omega_{obs},\psi_{obs},X_{obs},Y_{obs}\right]}^{T}$$ (28)

It has been confirmed that the velocity, yaw angle, and yaw rate of an obstacle can be measured using a camera, with a detection time of 40 ms, which is shorter than the verified AV control period of 50 ms [35]. By assuming that the velocity, yaw angle, and yaw rate of the obstacle can be measured, the discrete state space model of the obstacle can be constructed by referring to the discrete state space model of the AV. The predicted state of the obstacle is expressed as follows:

$$x_{obs}\left(k+n|k\right)=f^{k}\left(x_{obs}\left(k+n-1|k\right)\right)\cdot T_{s}\;+x_{obs}\left(k+n-1|k\right),1\leq n\leq p$$ (29)

According to the predictive state of obstacle and AV, the predictive potential field after fusing can be expressed as:

$$U\left(k+n/k\right)=g^{k}\left(x\left(k+n/k\right),x_{obs}\left(k+n/k\right)\right),\;1\leq n\leq p$$ (30)

During the obstacle avoidance process, the AV is required to accomplish three functions: avoiding obstacles, tracking paths, and maintaining velocity. Consequently, the cost function consists of three parts, as demonstrated below.

To reflect the fundamental obstacle avoidance capability and ensure stability for path tracking, the fused predictive potential field is utilized as a penalty term. At the sample time k, the penalty function is defined as follows.

$$J_{U,k}=\sum_{n=1}^{p}c_{4}\cdot U\left(k+n/k\right)$$ (31)

where *c*_4_ represents the weighting factor of the penalty function.

On the basis of obstacle avoidance, it is necessary to keep path tracking. In this paper, the local reference path is not re-planned, so the global reference path needs to be tracked. And it is defined as follow.

Building upon the obstacle avoidance capability, maintaining path tracking is also essential. It becomes necessary to track the reference path. This is defined as follows:

$$J_{Y,k}=||Y_{k}-Y_{ref,k}|{|}_{W}^{2}$$ (32)

where *Y*_*ref*,*k*_ represents the vector of y-coordinates for the global reference path within the predictive horizon, and *W* denotes the weighted matrix for path deviation.

Furthermore, it is essential to maintain the velocity to the greatest extent feasible.

$$J_{v,k}=||v_{x,k}-v_{ref,k}|{|}_{Q}^{2}$$ (33)

where $v_{x,k}$ corresponds to the longitudinal predicted velocity vector. $v_{ref,k}$ denotes the reference velocity vector. Additionally, *Q* signifies the weighted matrix associated with velocity deviation.

By taking into account the constraints of acceleration, yaw angle, and distance, the obstacle avoidance issue can be reformulated as the following optimization problem.

$$J_{k}=J_{U,k}+J_{Y,k}+J_{v,k}\;s.t.\;b_{l}\leq u\leq b_{u}$$ (34)

where $b_{l}=-[a_{ymax},a_{xmax}]$ and $b_{u}=[a_{ymax},a_{xmax}]$. By addressing the aforementioned optimization problem [35], the control variables *u* can be determined. The path can then be generated based on *u*, and path tracking can be carried out using the method described in [31].

## 4 Simulation

The simulation is carried out on the CarSim-Matlab co-simulation platform.

To evaluate and validate the performance of PFR-BPPS, the simulation results of PFR-BPPS will be compared with a potential field-based model predictive (PF-MP) method [36] and a moment-based model predictive (MB-MP) method [37]. In contrast to this study, reference [36] incorporates moving obstacles into the optimal control problem but does not account for velocity adaptability or path stability during path planning, and reference [37] can well realize obstacle avoidance in an uncertain environment with moving obstacles. Consequently, it is appropriate for assessing the distinctive features of the proposed method. This section employs three tests to verify the proposed approach. To emphasize different emergency situations, the trigger distance *d*_*t*_, which can be interpreted as the distance required to detect an obstacle, is specified for these tests. The configuration of the simulation scenarios is outlined in Table 3, and the negative sign indicates that the AV and the obstacle are moving in opposite directions.

**Table 3:** The setting of simulation scenarios.

Test	Test parameters	Result
Test 1	$v_{AV}$ =10m/s, $v_{obs}$ =-10m/s, *d*_*t*_=30 *m*	Figs 9–12
Test 2	$v_{AV}$ =20m/s, $v_{obs}$ =-20m/s , *d*_*t*_=45 *m*	Figs 13–16
Test 3	$v_{AV}$ =30m/s, $v_{obs}$ =-30m/s , *d*_*t*_=60 *m*	Figs 17–20

**Fig 9:**
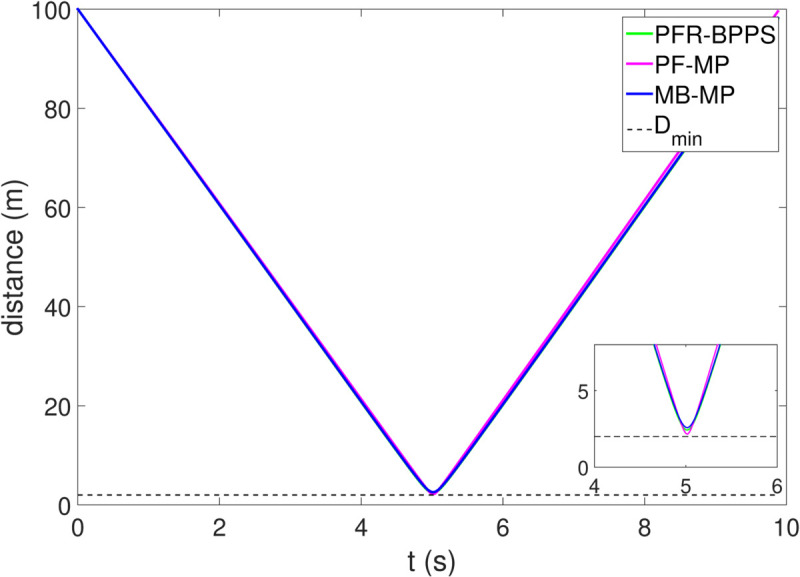
The distance between AV and obstacle in Test 1.

**Fig 10:**
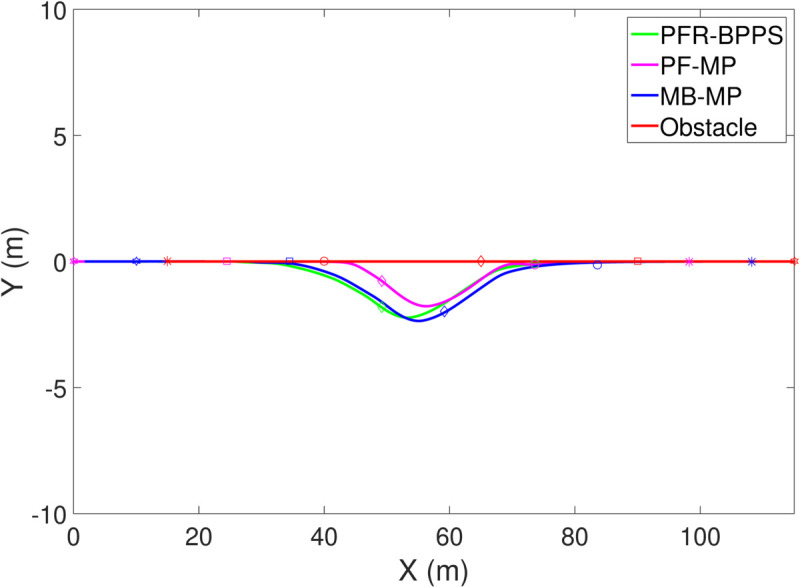
The track of AV and obstacle in Test 1.

**Fig 11:**
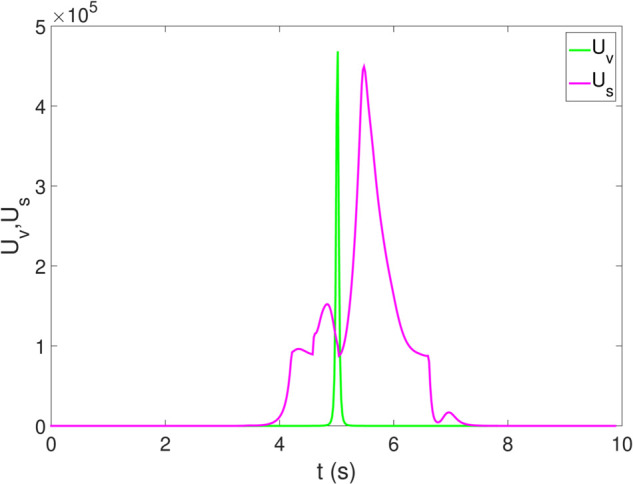
The change of $U_{v}$ and $U_{s}$ in Test 1.

**Fig 12:**
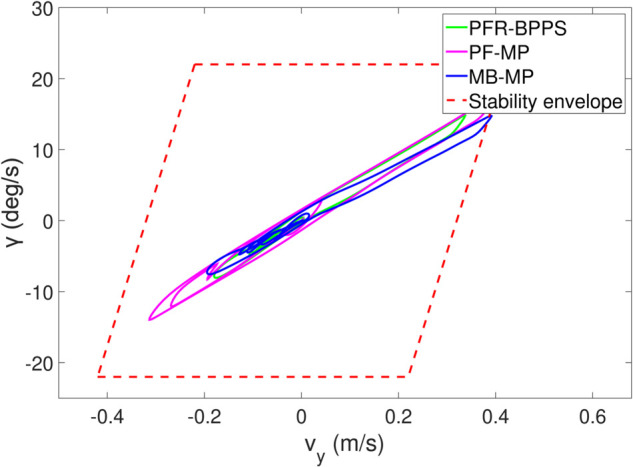
The change of γ and $v_{y}$ in Test 1.

*1) Test 1:* The outcomes of this test are illustrated in Figs 9–12. Fig 9 shows the changes in the distance between the obstacles and AV, and PFR-BPPS consistently remains greater than the predefined safe distance of 2 meters. Additionally, PF-MP and MB-MP generally maintain a safe distance as well. Fig 10 displays the trajectories of both the obstacle and the AV, where symbols with matching shapes represent their respective positions at the same time instances. Due to adapting to the velocity better, the path planned by PFR-BPPS makes the operation for obstacle avoidance earlier than that of PF-MP. Fig 11 displays the change of $U_{v}$ and *U*_*s*_ generated by PFR-BPPS. At the fifth second, $U_{v}$ suddenly increases, and then it decreases rapidly under the influence of potential velocity field, which helps AV avoid collision. *U*_*s*_ goes through a similar process, but it lasts much longer, which means it works for the stability of path at an earlier time. In Fig 12, *γ* and $v_{y}$ of PFR-BPPS are in the stability envelope, which makes the stability of AV completely controllable. Due to the relatively slow velocity of AV and the obstacle, *γ* and $v_{y}$ of PF-MP and MB-MP are also in the stability envelope.

**Fig 13:**
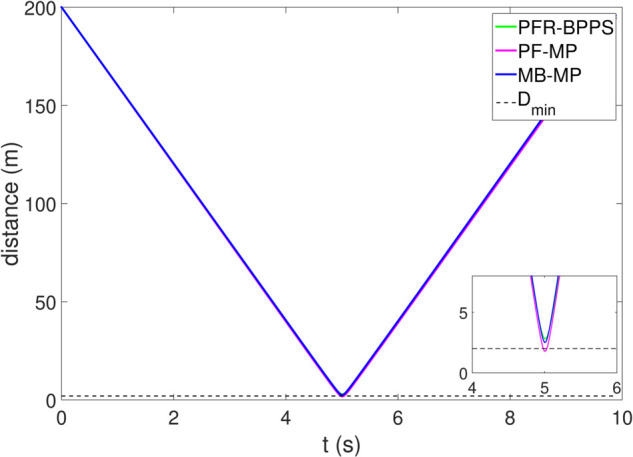
The distance between AV and obstacle in Test 2.

**Fig 14:**
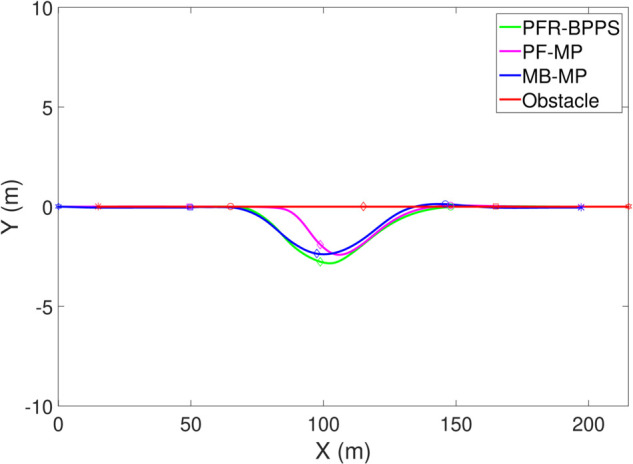
The track of AV and obstacle in Test 2.

**Fig 15:**
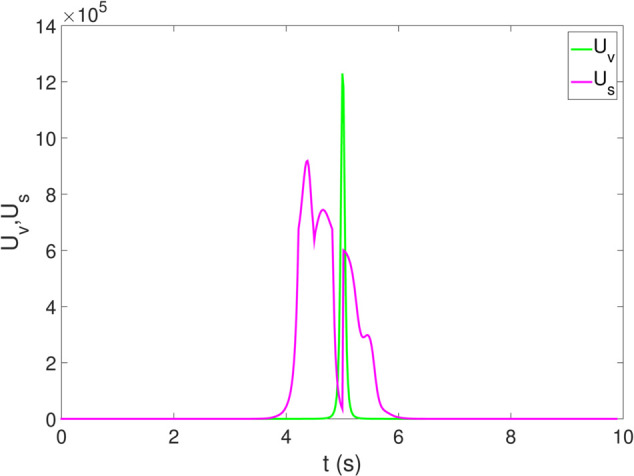
The change of $U_{v}$ and $U_{s}$ in Test 2.

**Fig 16:**
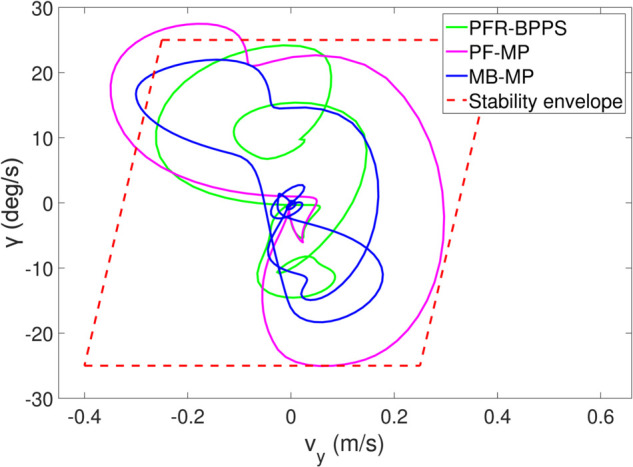
The change of γ and $v_{y}$ in Test 2.

*2) Test 2:* The outcomes of this test is displayed in Fig 13–16. In Fig 13, PFR-BPPS and MB-MP still keep a safe distance from obstacle, however, PF-MP can not adapt to a large relative velocity and the distance is no longer safe. In Fig 14, due to adapting to the velocity better, the path planned by PFR-BPPS makes the operation for obstacle avoidance earlier than that of PF-MP, which helps PFR-BPPS keep a safer distance. Fig 15 shows the change of $U_{v}$ and *U*_*s*_. The change of $U_{v}$ displays that the potential velocity field keeps AV in a safer velocity region. *U*_*s*_ is bigger than the one in test 1, because *γ* and $v_{y}$ in this test are approaching the boundary of stability envelope, however, as shown in Fig 16, PF-MP and MB-MP are out of the stability envelope, which may lead to instability of AV.

**Fig 17:**
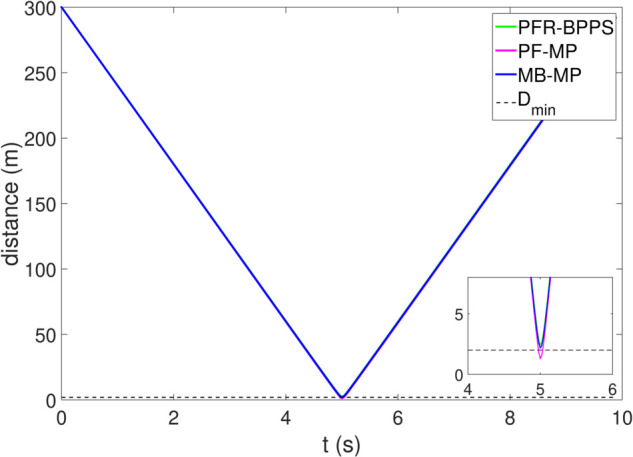
The distance between AV and obstacle in Test 3.

**Fig 18:**
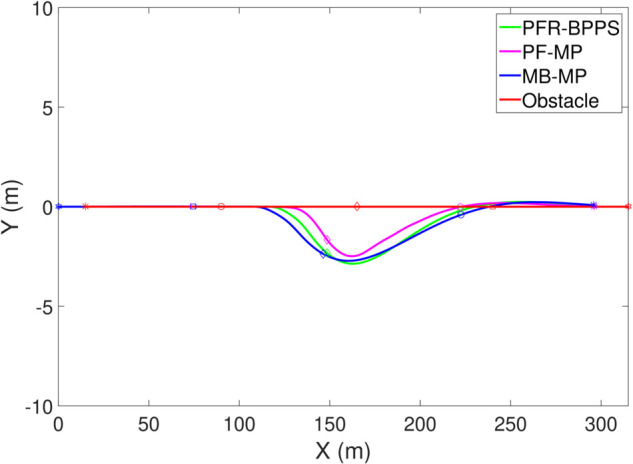
The track of AV and obstacle in Test 3.

**Fig 19:**
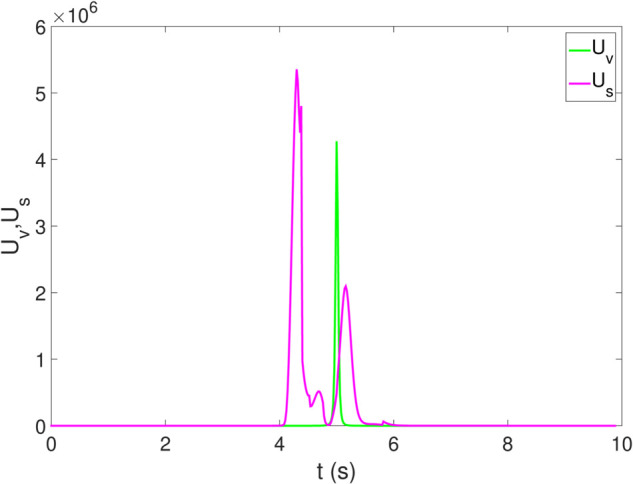
The change of $U_{v}$ and $U_{s}$ in Test 3.

**Fig 20:**
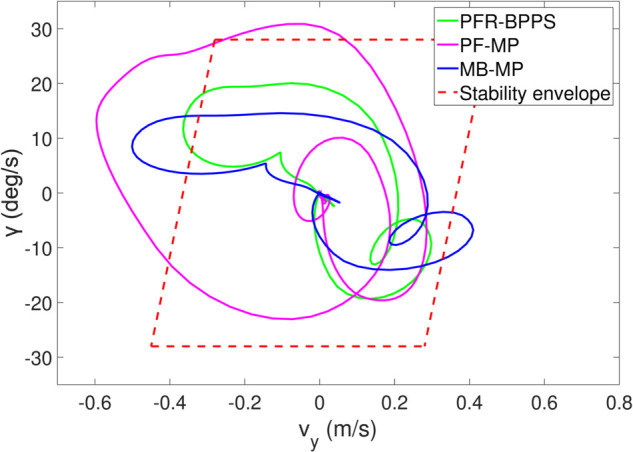
The change of γ and $v_{y}$ in Test 3.

*3) Test 3:* The result of test 3 is displayed in Figs 17–20. In Fig 17, PFR-BPPS and MB-MP still keep a safe distance from obstacle, however, PF-MP can not keep a safe distance, which lead to a collision with obstacle. In Fig 18, under the work of potential velocity filed, the path planned by PFR-BPPS makes the operation for obstacle avoidance earlier than that of PF-MP, which helps PFR-BPPS keep a safer distance. Fig 19 exhibits the change of $U_{v}$ and *U*_*s*_. The change of $U_{v}$ displays that the potential velocity field keeps AV in a safer velocity region. *U*_*s*_ is bigger than the one in test 2, even bigger than $U_{v}$, because *γ* and $v_{y}$ are out of stability envelope in this particular emergency scenarios, however, as shown in Fig 20, the range of these two parameters is still much smaller than that of PF-MP and MB-MP. This means that the stability of PFR-BPPS, which may be implemented in the path tracking layer, is much easier to control.

The above indicate that the proposed method improves both the adaptability of velocity and the stability of path under different emergency situations in path planning layer.

## 5 Conclusions

This paper proposes a potential field reconstruction-based path planning system (PFR-BPPS) for guaranteeing the stability of path in emergency scenario. In the path planning process, the adaptability of velocity has been enhanced by a potential velocity filed, and the stability of path are also considered through a potential stability field. In addition, an adaptive fusion module is designed to fuse these two potential fields adaptively in different emergency scenarios. The simulation is carried on the Carsim-Matlab co-simulation platform and the simulation result indicates that the path planned by PFR-BPPS has excellent performance for enhancing the adaptability of velocity and the stability of path in emergency scenario.

## Supporting information

PFR-BPPS_data. (XLSX)
